# Large Foreign Body as a Nidus for a Common Duct Stone in a Patient Without Spontaneous Biliary Enteric Fistula or Previous Abdominal Surgery

**DOI:** 10.1155/1993/51546

**Published:** 1993

**Authors:** Francesco Cetta, Francesco Lombardo, Stelio Rossi

**Affiliations:** 1 Institute of Surgical Clinic University of Siena viale Bracci Siena 53100 Italy; 2 Institute of Anatomy Interuniversity Center for Research on Hepatobiliary diseases Italy

## Abstract

We report a case of a brown pigment gallstone, which formed around a phytobezoar in the common bile duct, in a patient without spontaneous biliary enteric fistula or previous abdominal surgery. A brief comment on the possible origin of the phytobezoar in this case and on the pattern of deposition of brown material over a pre-existent nidus is also presented.


*HPB Surgery*, 1993, Vol. 6, pp. 235–243
Reprints available directly from the publisher
Photocopying permitted by license only

© 1993 Harwood Academic Publishers GmbH
Printed in the United States of America

### CASE REPORT

### LARGE FOREIGN BODY AS A NIDUS FOR A COMMON DUCT STONE IN A PATIENT WITHOUT SPONTANEOUS BILIARY ENTERIC FISTULA OR PREVIOUS ABDOMINAL SURGERY

FRANCESCO CETTA*, FRANCESCO LOMBARDO* and STELIO ROSSI**
*Institute of Surgical Clinic*; Institute of Anatomy**, University of Siena, Italy*
*Interuniversity Center for Research on Hepatobiliary diseases**

*(Received 13 August 1991)*

We report a case of a brown pigment gallstone, which formed around a phytobezoar in the common bile duct, in a patient without spontaneous biliary enteric fistula or previous abdominal surgery. A brief comment on the possible origin of the phytobezoar in this case and on the pattern of deposition of brown material over a pre-existent nidus is also presented.

KEY WORDS: Choledocholithiasis, foreign bodies, brown gallstones, calcium salt deposition

Foreign bodies or phytobezoars are not uncommon in the bile duct, either isolated or as a nidus for common duct stones in patients with previous biliary enteric anastomosis or sphincterotomy. This is probably due to the loss of the sphincteric mechanism, permitting the reflux from the alimentary tract into the bile ducts of indigested vegetable fibres or food particles[1–5]. On the contrary, phytobezoars are very rare in patients without biliary enteric communication.

A case is reported of a patient with phytobezoar acting as a nidus for a large common duct brown pigment stone. This patient did not have a spontaneous biliary enteric fistula, any previous trauma or intervention for abdominal surgery.

### CASE REPORT

A 72-year-old female patient with no previous abdominal trauma or surgery, was admitted to our Institution with multiple episodes of biliary pain during the last three years. She never had jaundice or cholangitis. Previous ultrasound examination and i.v. cholangiography showed dilated bile ducts and multiple stones both in the gallbladder and in the common duct. On admission, bilirubin, transaminases and alkaline phosphatase were normal, while amylasaemia was slightly increased

Address correspondence to: Prof. Francesco Cetta, Istituto di Clinica Chirurgica, Università di Siena, viale Bracci 53100, Siena, Italy

(500 Somogy units). At surgery, operative cholangiography, performed through a Caroli's cannula, inserted into a large cystic duct, showed the presence of a large stone impacted in the ampulla of Vater and of multiple smaller stones located more cranially (Figure 1). In particular, there was no direct or indirect sign of biliary-enteric or bilio-biliary fistula.

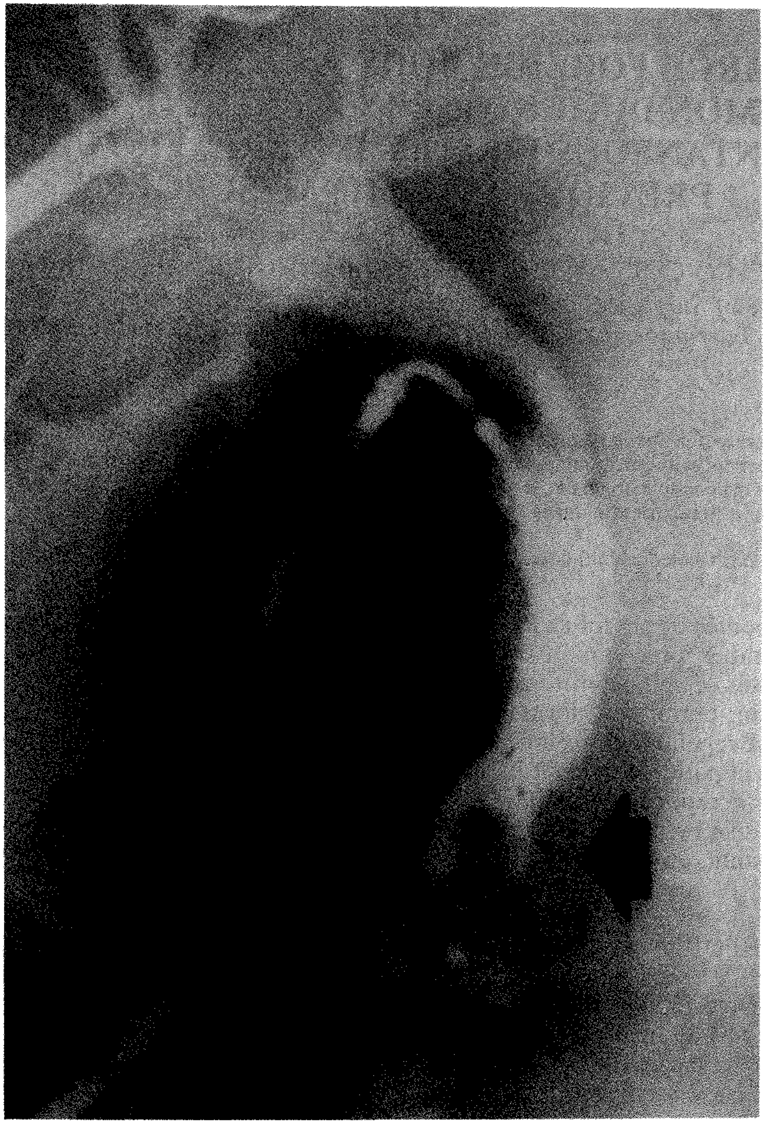

**Figure 1** Operative cholangiography showing the presence of a large stone impacted in the ampulla of Vater together with multiple stones of various size and morphology.

**Figure 2** Stones found in the common duct: the larger stone contained the phytobezoar; spheroidal stones were found in the lower third of the common duct, while faceted stones were found near the cystic duct. (*See colour plate at back of issue*).

Cholecystectomy, choledochoscopy and common duct stone removal, without sphincterotomy were performed. The common duct was drained through a T-tube, that was removed 12 days postcholecystectomy, after performing T-tube cholangiography, which confirmed the absence of bilio-biliary or biliary-enteric fistulas. The gallbladder, which was extremely distended, was entirely filled with faceted stones, 2–4 mm in size, similar to faceted stones found in the common duct (Figure 2). Culture of the common duct bile demonstrated the presence of E. coli (CFU $> 10^6$ per ml). Culture of the gallbladder bile also showed the presence of E. coli, but at lower concentration ($10^5$ CFU per ml). Light stereomicroscopy and scanning electron microscopy of the gallstones were performed. In particular, the large brown stone impacted in the ampulla, contained a phytobezoar (Figures 3–4). Stereomicroscopy clearly showed the mutual relationships between the indigested vegetable fibre, 3 cm long, and the various stone compounds. X-ray diffractometry and infrared spectroscopy[8,9] showed the presence of calcium bilirubinate (55% of stone dry weight), calcium palmitate (21%) and cholesterol (15%). Scanning electron microscopy showed the typical presence of bacteria inside the stone

**Figure 3** Cross section of the various types of common duct stones: (a) the stone containing the phytobezoar consisted entirely of brown pigment material; (b) the larger spheroidal stone had a nucleus similar to the smaller faceted stone and a brown periphery. (*See colour plate at back of issue*).

(Figure 5)[9–12]. Gallbladder wall and a small specimen of the common duct wall, both at histology and at transmission electron microscopy, showed the presence of chronic inflammation, with diffuse epithelium loss. Gallbladder stones contained cholesterol (85%), bilirubinate (5%) and carbonate (5%), without calcium palmitate.

Stones present in the common duct were of different types, according to their site: (a) *near the cystic duct*: similar to gallbladder stones; (b) *more caudally*: spheroidal or irregular, with a nucleus similar to gallbladder stones and a brown periphery (35% bilirubinate, 10% palmitate); (c) *in the ampulla*: the large brown stone, containing the phytobezoar.

The patient, included in a special group of patients with scheduled radiologic follow-up, also had ERCP 18 months after operation. The absence of a biliary-enteric fistula, even in the juxtapapillary region[13] was again excluded. The patient has had no symptoms in the last 2 years.

### COMMENT

The present case report requires comment on two areas.

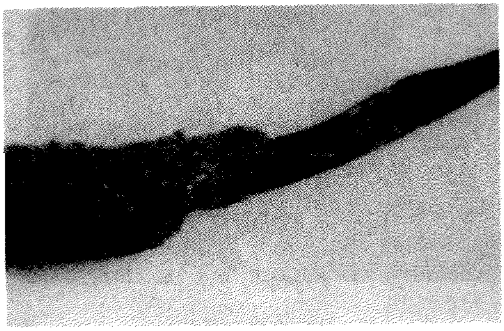

(a)

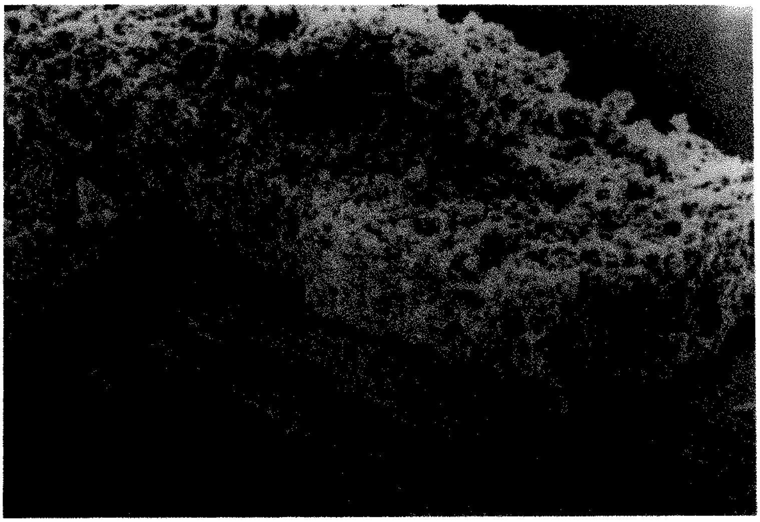

(b)

**Figure 4** *a*: Stereomicroscopy and *b*: scanning electron microscopy of the larger stone containing the phytobezoar, showing the mutual relationships between the indigested fibre and the various stone compounds.

*(1) Origin of the phytobezoar.* To explain the presence of a large indigested fibre inside the common duct in a patient with no previous trauma, spontaneous fistula or abdominal surgery, the following hypothesis can be suggested. Since the patient had multiple small stones, 2–4 mm in size, and a large cystic duct, it is likely that some stones passed from the gallbladder into the common duct and then into the duodenum, through the papilla of Vater. The sphincter of Oddi, as usually occurs after the spontaneous passage of a stone, remained hypotonic for a while, due to trauma, so permitting duodeno-biliary reflux both of the indigested vegetable fibre and of bacteria, that could be present in the duodenum, because of the age of the patient[10].

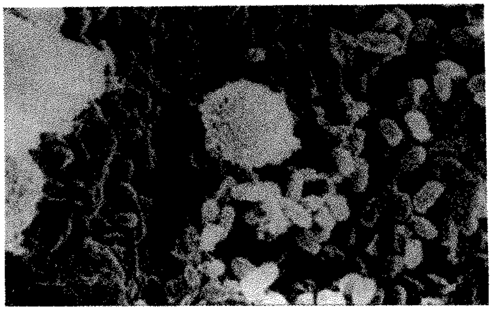

**Figure 5** Scanning electron microscopy showing the typical presence of bacteria inside the larger stone. Magnification$^{5000\times}$.

Subsequently, there was bacterial overgrowth, due to bile stasis, thus facilitating the precipitation of the typical components of brown stones (calcium bilirubinate and palmitate), both over the phytobezoar and over some other faceted stones of gallbladder origin, that had passed into the common duct.

*(2) Pattern of deposition of brown material over a preexistent nidus* (either a foreign body or a faceted stone of gallbladder origin).

We have previously suggested that carbonate and palmitate are mutually exclusive anions in gallstones: carbonate is typical of gallbladder stones, palmitate of primary common duct brown stones[7–11]. The present report also suggests that there is a gradient in the concentration of these salts in gallstones, depending on their site in the gallbladder[14,15] or in the common duct. After cystic duct obstruction, calcium carbonate precipitates in the gallbladder over previous cholesterol or mixed stones and calcium concentration is greater on the stone closer to the fundus and less on the stone impacted in the infundibulum[14,15]. We also have frequently observed similar findings in patients with porcelain gallbladder (8 cases in our series) or in some subjects with functionally excluded gallbladder. In these cases, stones usually were found firmly impacted in the infundibulum and forming a pouch in which they could have remained for a long time and grow without shifting their position[8–10]. Even if the occurrence of septa, pouches or multiple microenvironments is more frequent in the gallbladder, we have also observed 3 old patients[16] with no previous operation, who had single or multiple strictures in the common duct at variable distance from the sphincteric zone with no histologic sign of malignancy ("focal sclerosing cholangitis" according to Blumgart)[17]. Brown stones (containing calcium palmitate) were found cranially to or between these strictures in all cases. Two of these patients had brown stones only in the common duct with no stones in the gallbladder, while in the third case faceted cholesterol stones were concomitantly present in the gallbladder. In the present patient, there was no evidence of pouches or focal strictures, which could have subdivided the common duct into multiple microenvironments. Therefore, it can be suggested that common duct stones maintained their position only because of the mutual spatial relationships among the faceted stones (more than 15, Figures 2–3) coming from above through the cystic duct and the foreign body coming from below through the papilla of Vater. The latter also could have conditioned at some extent the position of stones located more caudally, limiting their movements. In addition, we have documented that brown stones larger than 2 cm can form in old patients, in the presence of bile stasis and infection, even within 2 months after cholecystectomy, in subjects with previous stones of other type (cholesterol), if a foreign body (suture material) acts as a nucleus[19,20]. Therefore, the time lapse preceding brown coat formation around an exogenous nucleus could be very short.

In contrast to calcium carbonate concentration in gallbladder stones after cystic duct obstruction, calcium palmitate concentration in common duct stones was maximal in the brown stone impacted in the ampulla containing the foreign body, and increasingly less on the faceted stones located more cranially, while it was null on the faceted stones that were found in the common duct near the cystic duct confluence. It can be hypothesized that, even if the extrahepatic bile tract is a ductal system with a uniform distribution of fluids (and then also with a uniform bacterial concentration), the presence of multiple stones or foreign bodies facilitates the occurrence of multiple microenvironments, each one with different bacterial concentrations and physical chemical conditions. This variability, together with the different stay of stones in the ducts, could be responsible for the variable precipitation of pigment material on stones primarily formed in the gallbladder. Concentration of palmitate and bilirubinate in common duct stones with a nucleus different from the periphery was greater near the ampulla or immediately above a common duct stricture and increasingly less, moving towards the main hepatic confluence, not only in the present case, but also in 11 other patients with bile infection observed in the course of a prospective study[7–10].

In conclusion, the present report shows a very unusual situation, that can be regarded just as a rarity. However, if we add the present findings to our cumulative data on gallstone pathophysiology[6–11,17–20], it can also be suggested that pathogenic factors which, together with bile stasis, determine calcium salt deposition on gallstones: (i) are various; (ii) facilitate the precipitation of different calcium salts in the gallbladder and in the common duct; (iii) are more active in the gallbladder fundus for calcium carbonate and near the ampulla of Vater for palmitate. These statements need to be confirmed by others. However, evidence is accumulating in this direction and the present case report with its peculiarity is undoubtedly in favor of the suggested hypothesis.

*Acknowledgement*

The present research has been supported by a grant from the Italian National Research Council (CNR): Grants No. 89.02491, No. 90.01446.CT04, No. 91.00236.CT04.

### *References*

1. Ban, J.L., Hirose, F.M. and Benfield, J.R. (1972) Foreign bodies of the biliary tract: report of two patients and a review of the literature. *Ann. Surg.*, **176**, 102–107
2. Bedogni, G. *et al.* (1986) Foreign bodies of the biliary tract, endoscopic management. *Dig. Dis. Sci.*, **31**, 1100–1104
3. Conroy, B. and Metcalf, M.J. (1982) Choledocholithiasis associated with plant material in the common bile duct. *J.R. Coll. Surg. Edinb.*, **27**, 244–245
4. Orda, R., Leviav, A., Ratan, I., Stadler, J. and Wiznitzer, T. (1986) Common bile duct stone caused by a foreign body. *J. Clin. Gastroenterol.*, **8**, 466–468
5. Janson, J.A. and Cotton, P.B. (1990) Endoscopic treatment of a bile duct stone containing a surgical staple. *HPB Surg.*, **3**, 67–71

6. Cetta, F., Tanzini, G., Palasciano, G., Petracci, M., Calfa, C. and Cappelli, A. (1988) Postcholecystectomy syndrome due to stones associated with a long cystic remnant or containing non absorbable suture material. *Proceed. XXVI World Congr. Int. Coll. Surg.*, **VI**, 229–234, Monduzzi Ed. Bologna
7. Cetta, F. (1989) Classification, composition and significance of common duct stones after cholecystectomy. In Gozzetti ed. "*Proceed. 1st Congr. Ital. Chap. HPB Surg.*" Esserre Publ., Bologna, 289–299
8. Cetta, F., Tommasini, E. and Saggese, N. (1985) Physicochemical analysis of pigment biliary stones. *Chir. Epatobil.*, **4**, 173–182
9. Cetta, F. (1986) Bile infection documented as initial event in the pathogenesis of brown pigment biliary stones. *Hepatology*, **6**, 482–489
10. Cetta, F. (1991) The role of bacteria in pigment stone disease. *Ann. Surg.*, **213**, 315–326
11. Cetta, F., Lombardo, F., Gargano, V. and Malerba, M. (1991) Calcium carbonate solubility in human common duct bile — Calcium carbonate precipitation in gallstones. *Gastroenterology*, **101**, 280–281
12. Stewart, L., Smith, A.L., Pellegrini, C.A., Motson, R.W. and Way, L.W. (1987) Pigment gallstones form as a composite of bacterial microcolonies and pigment solids. *Ann. Surg.*, **206**, 242–250
13. Ikeda, S. and Okada, Y. (1975) Classification of choledochoduodenal fistula diagnosed by duodenal fiberscopy and its etiological significance. *Gastroenterology*, **69**, 130–137
14. Phemister, D.B., Aronsohn, H.G. and Pepinsky, R. (1939) Variations in the cholesterol, bile pigment, and calcium contents of stones formed in gallbladder and in the bile ducts with degree of associated obstruction. *Ann. Surg.*, **109**, 161–186
15. Phemister, D.B., Rewbridge, A.G. and Rudisill, H.J. (1931) Calcium carbonate gallstones and calcification of the gallbladder following cystic duct obstruction. *Ann. Surg.*, **94**, 493–516
16. Cetta, F., Lombardo, F., Giani, S., De Martino, A., Testi, W., Gallorini, M., Vegni, G., Cappelli, A. and Civitelli, S. (1990) "Colangite sclerosante focale ed altre stenosi localizzate benigne non iatrogene della via biliare principale." *Proceed. Congr. SIRC- SIFIPAC* Monduzzi Publ. Bologna, 335–341 (in Italian)
17. Smadja, C., Bowley, N.B., Benjamin, I.S. and Blumgart, L.H. (1983) Idiopatic localized bile duct strictures: relationship to primary sclerosing cholangitis. *Am. J. Surg.*, **146**, 404–408
18. Cetta, F., Tonelli, F. and Armenio, S. (1992) Postcholecystectomy common duct stones associated with non absorbable suture material. Composition, frequency and possible pathogenesis. *Hepatology*. Submitted for publication
19. Cetta, F., Lombardo, F., Sianesi, M. and Tonelli, F. (1992) Pathologic and clinical significance of foreign bodies in the bile tract: report on 64 gallstones containing or associated with foreign bodies analyzed in a single center. *Ann. Surg.* Submitted for publication
20. Cetta, F., Lombardo, F. and Guidoni, E. (1992) Intraparietal and intraluminal black pigment microstones associated with "pure" cholesterol stones. Pathogenetic, diagnostic and clinical importance. *Gastroenterology*. Submitted for publication

*(Accepted by S. Bengmark 19 December 1991)*

